# Vaccination rates in rheumatic diseases: a cross-sectional register study on the role of patient beliefs and physician engagement

**DOI:** 10.1093/rheumatology/keag123

**Published:** 2026-03-15

**Authors:** Samia Mehouachi, Denis Mongin, Gilles Eperon, Delphine S Courvoisier, Kim Lauper

**Affiliations:** Division of Rheumatology, Geneva University Hospitals, Geneva, Switzerland; Division of Rheumatology, Geneva University Hospitals, Geneva, Switzerland; Geneva Centre for Inflammation Research, Faculty of Medicine, University of Geneva, Geneva, Switzerland; Geneva Centre for Inflammation Research, Faculty of Medicine, University of Geneva, Geneva, Switzerland; Division of Tropical and Humanitarian Medicine, Geneva University Hospitals, Geneva, Switzerland; Division of Rheumatology, Geneva University Hospitals, Geneva, Switzerland; Geneva Centre for Inflammation Research, Faculty of Medicine, University of Geneva, Geneva, Switzerland; Division of Rheumatology, Geneva University Hospitals, Geneva, Switzerland; Geneva Centre for Inflammation Research, Faculty of Medicine, University of Geneva, Geneva, Switzerland

**Keywords:** autoimmune inflammatory rheumatic diseases, vaccination uptake, vaccination behavior, vaccination importance, immunization

## Abstract

**Objectives:**

To evaluate the vaccination attitude and behaviour of patients with autoimmune inflammatory rheumatic diseases (AIIRD), and identify factors associated with vaccination status, utilizing data from a national register.

**Methods:**

A cross-sectional survey was developed to assess behaviour around vaccination of patients with AIIRD, nested within the Swiss Clinical Quality Management (SCQM) register. The primary outcome was vaccination uptake evaluated through three parameters: checking vaccination status (i.e. ensuring that vaccinations were checked by a health professional), influenza and pneumococcal vaccination coverage. The main exposure evaluated was patient’s beliefs about vaccination importance and safety. Vaccination uptake was analysed using descriptive statistics, and associations with exposures using logistic regression. Missing data were imputed by multiple imputation, and multivariable analyses were adjusted for age, gender, body mass index, disease type, disease duration, treatment.

**Results:**

Of 2446 patients (59% women, 79% <65 years old) included, 52% believed in both the importance and safety of vaccine and 2% believed both that vaccination was neither safe nor important. Most patients (64%) reported no change in their vaccination beliefs after the COVID-19 pandemic. Among respondents, 48% had checked their vaccination status in the past 24 months, 51% reported vaccination against influenza and 33% against pneumococcal pneumonia. Discussions with the rheumatologist about vaccination during follow-up or before initiating treatment were positively associated with vaccination.

**Conclusion:**

Vaccination uptake was relatively low in this vulnerable population. Strategies promoting discussion with the rheumatologist about vaccination and before treatment could play a pivotal role in improving vaccination uptake among patients with AIIRD.

Rheumatology key messages

- Vaccination uptake is low among patients with autoimmune inflammatory rheumatic diseases despite perceived importance.
- Professional discussions significantly impact vaccination behaviours in autoimmune inflammatory rheumatic disease patients.

## Introduction

Autoimmune inflammatory rheumatic diseases (AIIRD) such as rheumatoid arthritis (RA), axial spondyloarthritis (axSpA) and psoriatic arthritis (PsA) are chronic inflammatory pathologies affecting the immune systems of patients. These conditions can lead to severe pain, joint damage, systemic complications and significant impairment in the quality of life. Over the past few decades, numerous treatments, especially disease-modifying anti-rheumatic drugs (DMARDs), have been developed, significantly improving the quality of life, function and even survival of these patients [1].

Although these treatments are beneficial, they also have an immunosuppressive effect [2, 3]. In addition, patients with AIIRD have probably an increased risk of developing infections secondary to the dysimmunity itself and the comorbidities often associated with the diseases [3–5]. The combination of dysimmunity, comorbidities and the use of these treatments therefore make this population more vulnerable to infections and at greater risk of developing severe forms of infections [2, 5,6]

Vaccination plays a crucial role in preventing infections [7, 8]. However, low vaccination uptake and poor adherence are often observed in this specific population [5, 9, 10]. Several factors, such as patients’ perception of vaccine safety or the lack of discussions on this topic with treating rheumatologists, could explain these low rates [4, 14, 15]. International societies such as the European League Against Rheumatism (EULAR) have issued recommendations, including the need to check vaccination coverage annually and to get vaccinated against influenza and pneumococcal infections [11]. Yet, we have limited data on vaccination uptake, the adequacy of vaccination schedules and patients’ attitudes toward vaccination in this specific population.

The aim of our study was to explore and evaluate the belief, attitude and behaviour related to vaccination among patients with three types of AIIRD (rheumatoid arthritis, axial spondyloarthritis and psoriatic arthritis). Data from a national register in Switzerland were used to assess vaccination uptake, beliefs about vaccination and factors associated with vaccination uptake.

## Methods

### Study population

This study is a nested cross-sectional survey conducted among patients diagnosed with rheumatoid arthritis, axial spondyloarthritis or psoriatic arthritis and followed in the longitudinal Swiss Clinical Quality Management register (SCQM). This register is a prospective register aiming to improve quality of care and actively contributing to research. Visits in the register range from one to four visits per year, providing a longitudinal perspective on their health and treatments. The data collection involves both rheumatologists and patients, from diverse settings (university and non-university hospitals, as well as private practices). Patients without information on the main exposures or outcomes were excluded (see below for details on exposures and outcomes).

### Data collection

Data collection for this study was carried out using a structured questionnaire (Supplementary Table S1) administered between 2021 and 2023, filled by the patients once, collecting information on:

- Vaccination status: vaccination status checked in the past 24 months by a health professional, and vaccination coverage of certain diseases such as influenza and pneumococcal infection.
- Beliefs: beliefs about vaccines and the possible impact of the COVID-19 pandemic on vaccine attitude.
- Vaccination monitoring: discussions on the theme of vaccination with the treating rheumatologist during a visit, before the implementation of a treatment or a travel plan.

In parallel, during the same period, referring rheumatologists were asked whether they had checked the vaccination status of their patients in the past 24 months.

### Outcome

The main outcome was the evaluation of vaccination uptake evaluated using three distinct self-reported parameters:

- Vaccination status checked in the past 24 months.
- Influenza vaccination coverage.
- Pneumococcal vaccination coverage.

Patients could answer ‘yes’, ‘no’ or ‘unknown’. Unknown was classified as ‘no’ for the main analysis.

### Exposures of interests

We initially planned to include two main exposures of interest related to vaccination beliefs:

- Belief in the importance of vaccination, assessed with the question:

‘Do you think it is important, that everyone get the recommended vaccines for themselves and their children?’

Responses were categorized as ‘agree’, ‘no opinion’ and ‘disagree’.

- Belief in vaccine safety assessed with the question: ‘Do you think the vaccines are safe for you?’

Responses were categorized as ‘not safe’, ‘fairly safe’, ‘safe’.

However, due to strong collinearity between the two variables, only perceived importance of vaccination was retained as the main exposure in the final multivariable models.

### Other covariates of interests

Other exposures of interest collected through the questionnaire included the following questions:

- ‘Did you discuss vaccination with your rheumatologist?’
- ‘Was it checked that your vaccinations were up to date before the introduction of your current rheumatological treatment?’
- ‘Have you discussed with a doctor the vaccinations needed for future travel before starting your current rheumatologic treatment?’

For all these questions, the possible answers were ‘Yes’, ‘No’ and ‘I don’t know’. The ‘I don’t know’ were classified as ‘No’ for the main analysis.

In addition, participants were asked about their desire for vaccination since the COVID-19 pandemic with responses categorized as ‘Less’, ‘Equal’ and ‘More’.

Data collected through the register were age (< or > 65 years old), gender, type of disease, disease duration, treatments (biological and targeted-synthetic disease-modifying antirheumatic drug (b/tsDMARD), conventional synthetic DMARD (csDMARD) or no DMARDs), glucocorticoid use (Yes/No), Body Mass Index (BMI), categorized in normal weight (<24.9), pre-obesity (between 25 and 29.9) and obesity (30 or more).

### Ethics

All patients included have signed a consent to the use of their data for research purposes. The data used in this study were collected in accordance with ethical principles and data protection standards. All procedures were approved by the Human Research Ethics Committee of Geneva (Approval No. 2023-00894). Data were deidentified and were stored in a secure database accessible only to authorized researchers.

### Statistical analysis

Proportions and frequencies were reported for categorical variables. Means, standard deviation, median, and first and third quartiles were used to describe continuous variables. Multivariable analysis was conducted using logistic regression. Three logistics models were built for the three distinct main outcomes (checked vaccination status, influenza coverage and pneumococcal coverage). The main exposures evaluated initially were beliefs regarding importance of vaccination and safety of vaccination. Due to the strong correlation between these two exposures (Supplementary Table S2), only the perceived importance was kept in the models. The other independent variables (covariates of interests) were selected based on their presumed association with vaccinations outcomes. Missing data were imputed using multiple imputation with chained equation with 20 samples, and predictive mean matching for continuous variables and logistic regression for categorical variables. The significance level was set at 5%. All statistical analysis were conducted using R software (Version 4.3.1).

### Sensitivity analysis

To evaluate the impact of uncertain responses (category ‘I don’t know’ provided in the three outcomes and in independent covariates of interests) on our results and test the robustness of our analysis, we considered these responses as missing data in a sensitivity analysis, which were then imputed with other missing covariates.

## Results

### Population characteristics

Of the 2446 patients included (Table 1), 37% had rheumatoid arthritis, 39% axial spondyloarthritis and 24% psoriatic arthritis. With respect to treatments, 72% were treated with bDMARDs, 17% had no DMARDs and 6% were taking glucocorticoids. The proportion of patients without DMARD treatment was largely driven by those with axial spondyloarthritis and psoriatic arthritis, who may be managed with NSAIDs only. In contrast, most patients receiving glucocorticoids had rheumatoid arthritis, in which the proportion without DMARD treatment was much lower (Supplementary Table S3). Most patients (59%) were female, with a majority (79%) being under the age of 65.

**Table 1:** Population characteristics.

Variables	Level	Overall (N (%))	Missing data (N (%))
**N**		**2446**	**2446**
**Age**	≤65	1942 (79)	0 0 (0.0)
**Gender**	Female	1455 (59)	0 0 (0.0)
**Body Mass Index^a^**	Normal weight	1005 (45)	209
	Pre-obesity	773 (35)	
	Obesity	459 (21)	
**Disease**	Rheumatoid arthritis	900 (37)	0 0 (0.0)
	Axial spondyloarthritis	942 (39)	
	Psoriatic arthritis	604 (25)	
**Disease duration**			
(Median [IQR])		11.00 [5, 17]	47
**Treatments**	b/tsDMARD^b^	1758 (72.0)	0 0 (0.0)
	csDMARD^c^	260 (11)	
	No DMARD	428 (17)	
**Glucocorticoids**	Yes	151(6.2)	

a Normal weight: <25kg/m^2^, Pre-Obesity: 25–29.9 kg/m^2^, Obesity: >30 kg/m^2^.

b Biological and targeted-synthetic disease-modifying antirheumatic drug.

c Conventional synthetic disease-modifying antirheumatic drug.

Most patients believed that receiving recommended vaccines for themselves and their children was important, with only 3% disagreeing. Regarding the belief of vaccine safety, 55% of participants considered vaccination safe, 36% rather safe and 9% unsafe (Table 2). Taken together, 52.2% believed both in the importance and safety of vaccines, while 2.4% believed it was not important to follow the recommendation and that vaccines were unsafe (Supplementary Table S2).

**Table 2:** Summary of self-reported vaccination uptake, beliefs, attitude and behaviours.

Variables	Level	Overall (NA^a^ included)
Total population		2446
Outcomes		
Vaccination status checked in the last 24 months	Yes	1164 (48.0)
	No	654 (27)
	No answer	628 (26)
Influenza coverage	Yes	1254 (51)
	No	1156 (47)
	No answer	36 (1.5)
Pneumococcal coverage	Yes	812 (33)
	No	732 (30)
	No answer	902 (37)
Exposures		
Main exposures of interest		
Vaccination importance	Disagrees	72 (2.9)
	No opinion	478 (20)
	Agrees	1896 (78)
Vaccination safety	Not safe	233 (9.1)
	Fairly safe	889 (36)
	Safe	1334 (55)
Other exposures of interest		
Vaccination ever discussed with rheumatologist	Yes	
	No	1945 (80)
	No answer	
Vaccination status verified before treatment	Yes	1429 (58)
	No	1017 (42)
	No answer	
Vaccination discussed travel requirements	Yes	656 (27)
	No	1790 (73)
	No answer	
Vaccination desire since COVID-19	Less	440 (18)
	Equal	1562 (64)
	More	444 (18)
	No Answer	

a Not available.

During the study period, 80% of patients reported having discussed vaccination with their rheumatologist, 58% did so before initiating their current treatment and 27% had addressed this topic before considering travel plans (Table 2). The COVID-19 pandemic had a variable effect on patients, with 18% indicating that it increased their desire to be vaccinated but also 18% reporting a decreased desire to be vaccinated (Table 2). During this period, 31% of rheumatologists checked the vaccination status of their patients.

### Vaccination uptake in patients with AIIRD

**Figure 1:**
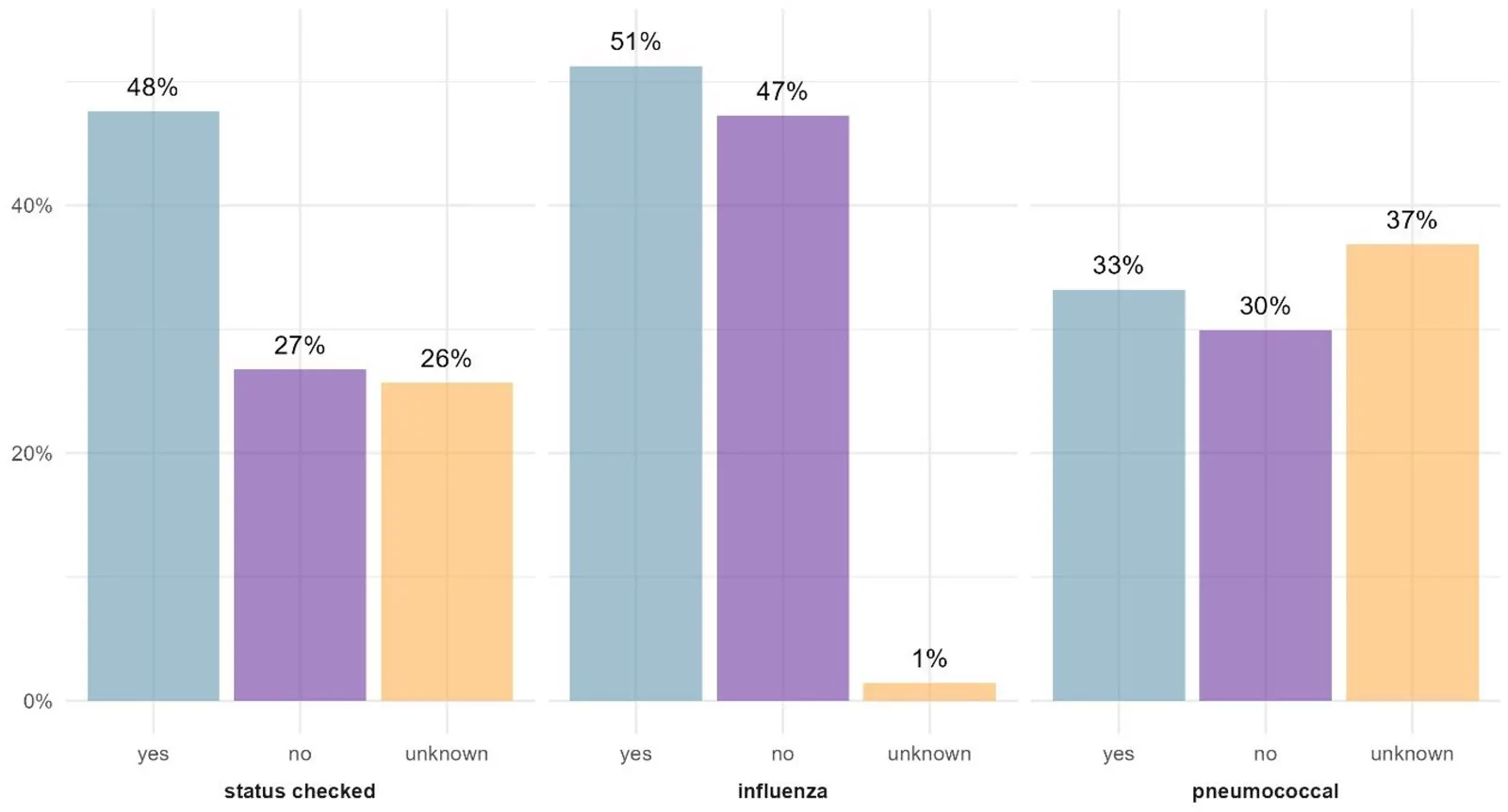
Proportion of participants whose vaccination status was checked by health professional in the past 24 months, influenza uptake and pneumococcal uptake

Almost 48% of patients reported having their vaccination status checked over the past 24 months (Table 2, Fig. 1). The reported influenza vaccination uptake for the 2020–2021 season was 51%, with very few people answering ‘unknown’ (1.5%, Fig. 1). The pneumococcal vaccination rate was 33%, with 37% not knowing their pneumococcal vaccination status (Fig. 1).

### Factors associated with vaccination uptake

#### Vaccination status checked in the past 24 months

**Figure 2:**
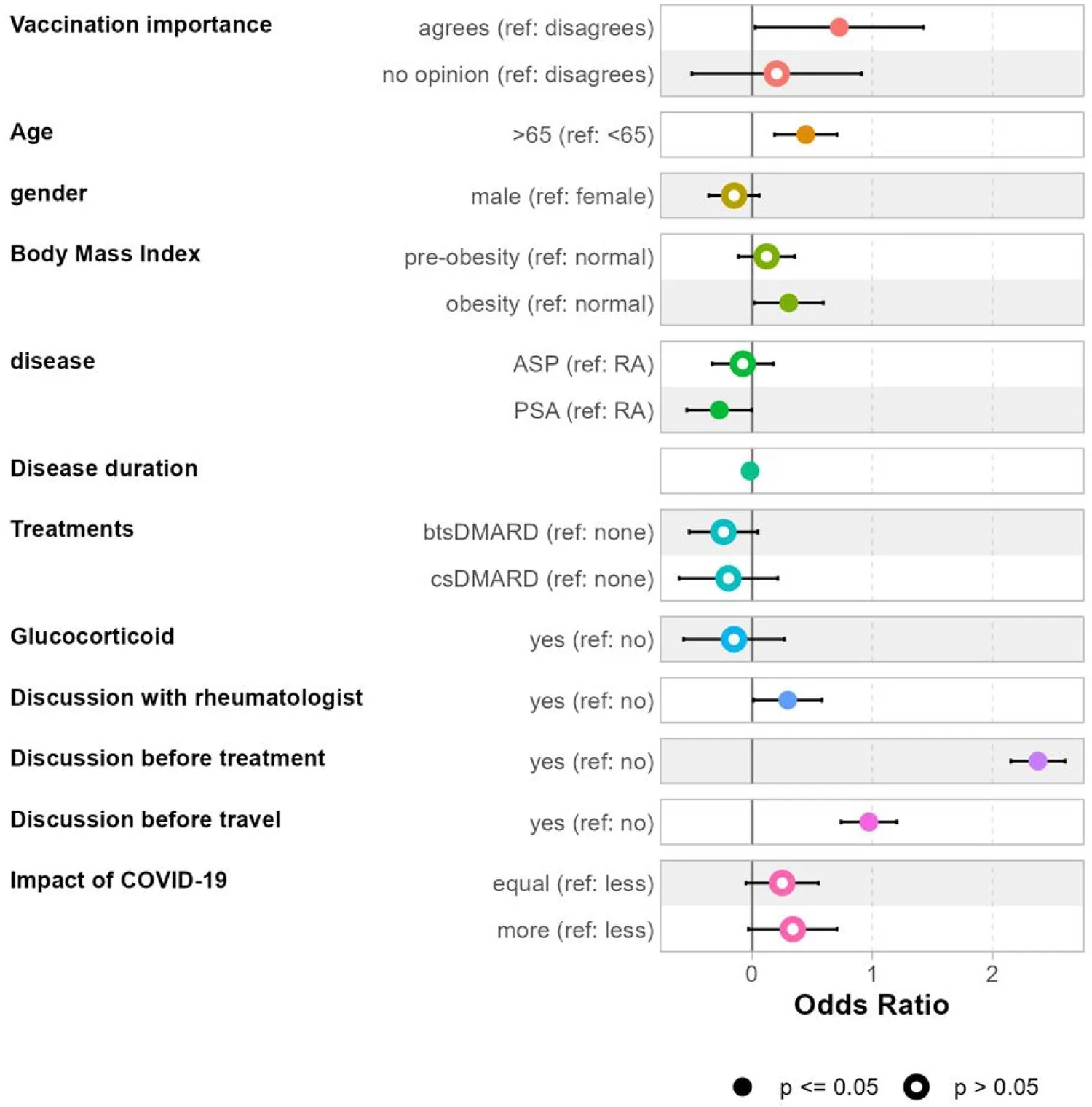
Vaccination status update in the past 24 months and factors associated in multivariate analysis

Perceived importance of vaccination [odds ratio (OR)=2.07, 95% confidence interval (CI) (1.03–4.16)] was significantly associated with this outcome (Fig. 2, Supplementary Table S4). Other factors associated with checking the vaccination status were disease type [patients with psoriatic arthritis less often had checked their vaccination status than patients with rheumatoid arthritis: OR = 0.76, CI 95% (0.58–1.00)], older age, obesity and shorter disease duration. Verifying vaccination status before initiating treatment, discussing vaccination with a rheumatologist and before initiating traveling, increased vaccine desire since the COVID-19 pandemic, was also associated with the outcome, but not the type of treatment (Fig. 2).

#### Influenza coverage

**Figure 3:**
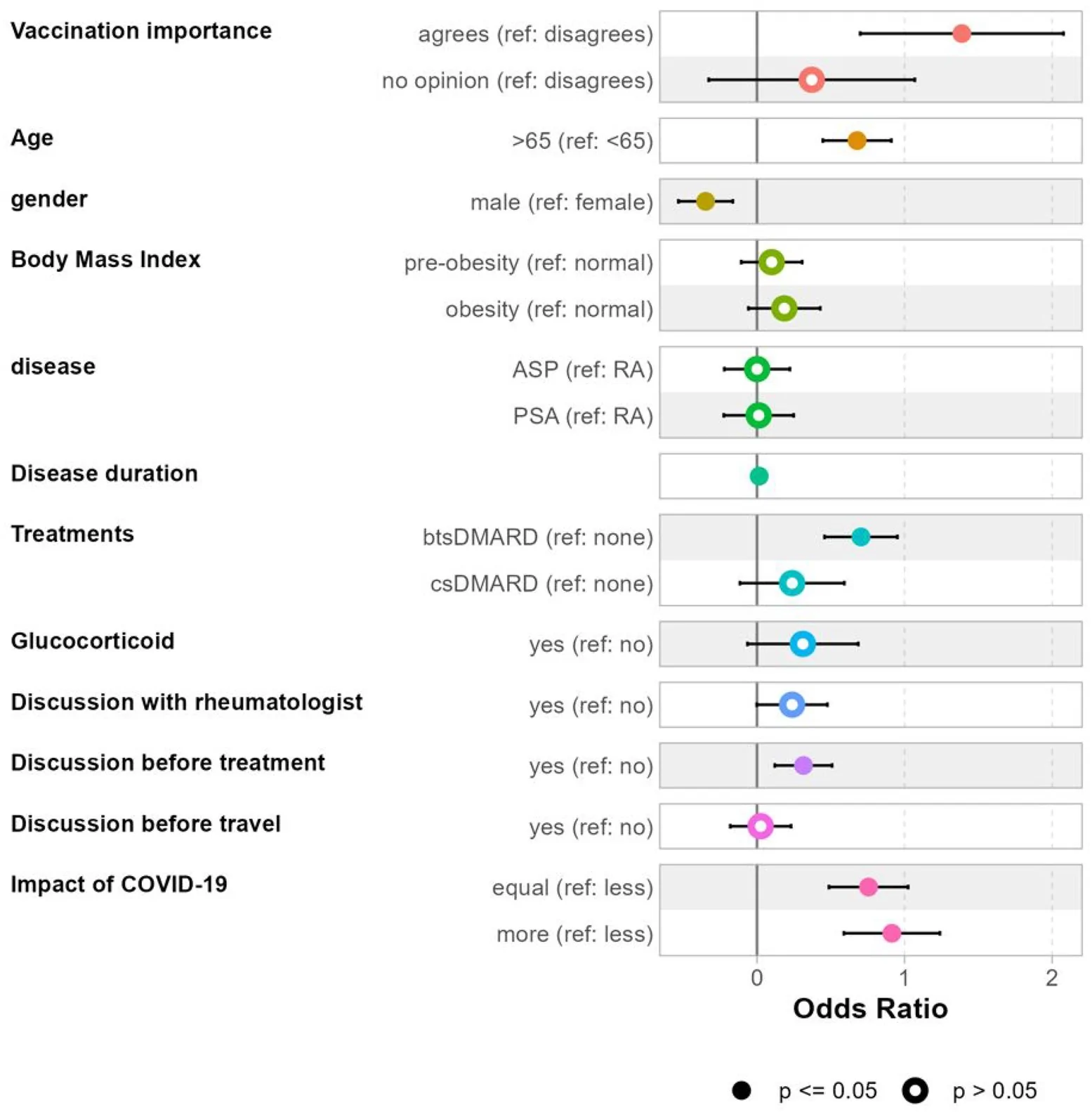
Factors associated in multivariate analysis with influenza uptake

Perceived importance of vaccination was associated with higher influenza vaccination coverage [OR=4.01, CI 95% (2.01, 7.98), Supplementary Table S5]. Age >65 years, being a man, disease duration, bDMARDs treatment, prior discussion with a rheumatologist about vaccination [OR=1.27, CI 95% (1.00, 1.61)], discussion about vaccination before initiating treatment and equal or increased desire to be vaccinated since the COVID-19 pandemic showed a positive association with influenza vaccination coverage (Fig. 3). Disease type did not influence influenza vaccination.

#### Pneumococcal coverage

**Figure 4:**
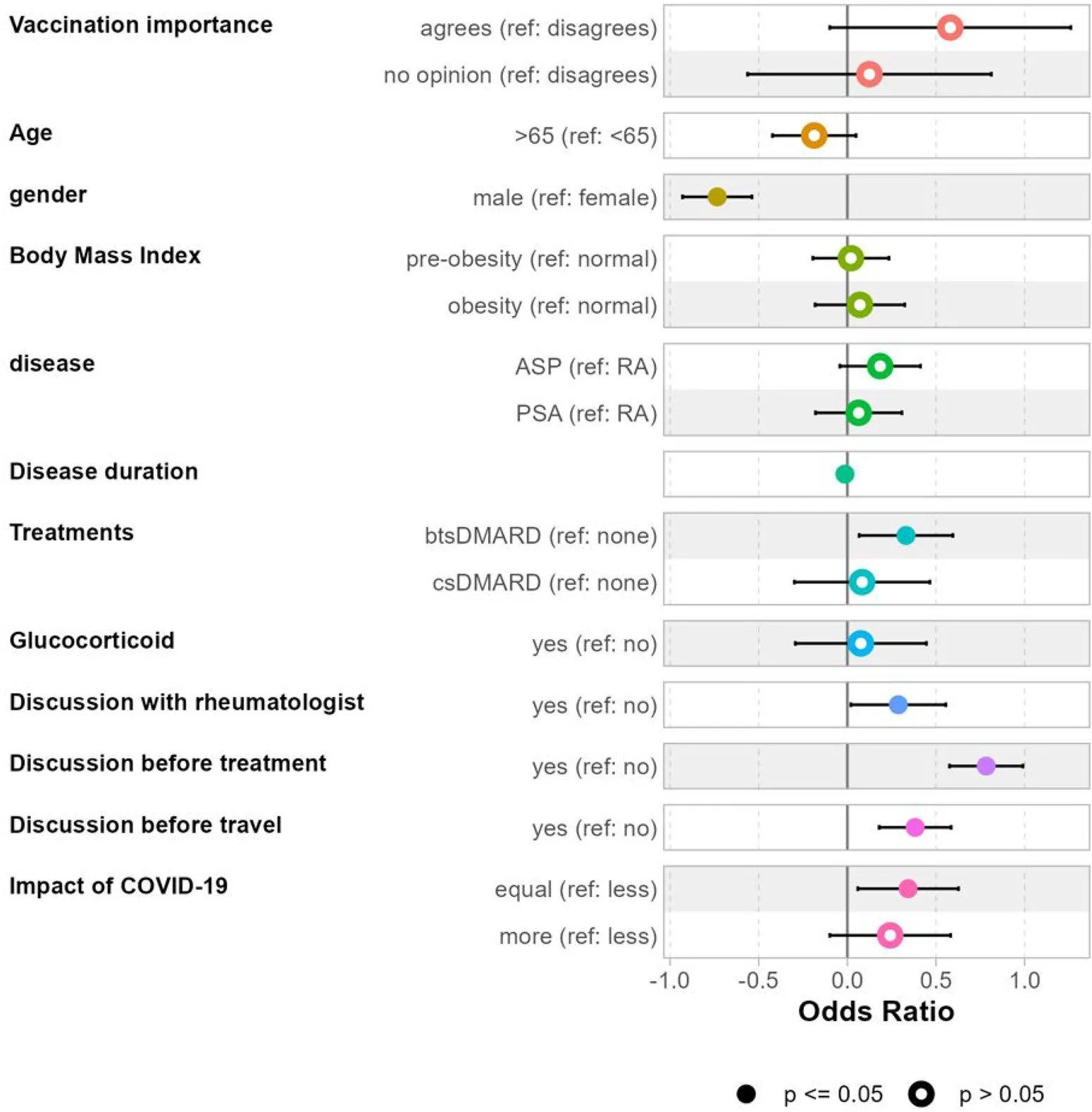
Factors associated in multivariate analysis with pneumococcal uptake

Perceived importance of vaccination was not associated with pneumococcal coverage (Fig. 4) (Supplementary Table S6). Prior discussion with a physician regarding vaccination [OR=1.33, CI 95% (1.02, 1.74)], discussion before initiating treatment and before travel, bDMARDs treatment and equal desire to be vaccinated since the COVID-19 pandemic were associated with increased vaccination uptake against pneumococcus (Fig. 4). Male gender and disease duration were negatively associated with pneumococcal vaccination coverage. Disease was not associated with pneumococcal coverage.

For all three outcomes, results of the sensitivity analyses, in which ‘I don’t know’ responses were treated as missing values and imputed using multiple imputation rather than recoded as ‘No’, were largely consistent with the main analysis (Supplementary Tables S7, S8 and S9).

## Discussion

Among the 2446 patients, most patients were female, with a majority being under the age of 65. Most patients considered vaccination important (78%), with 55% deeming it safe and 36% finding it fairly safe. A discussion on the topic of vaccination was reported by 80% of patients. Although 64% of patients felt that the COVID-19 pandemic had not significantly affected their desire to be vaccinated, those expressing a greater desire for vaccination since had increased vaccination uptake.

Overall, reported checks of the vaccination status in the past 24 months were 48% and specific vaccination was 51% for influenza and 33% for pneumococcus, in line with studies finding suboptimal vaccination coverage in this population, as well as lower vaccination rates against pneumococcus than against influenza [4, 9, 14–17]. Compared with the World Health Organization target of 75% vaccination coverage for influenza in at-risk populations, the overall vaccination uptake in Switzerland remains low. However, when compared with the general population, vaccination rates among at-risk groups are relatively higher. Between 2007 and 2017, self-reported influenza vaccination coverage in Switzerland was 16% in the general population and ranged from 36% to 48% among individuals aged over 65 years (Supplementary Table S10). These figures highlight that, while coverage remains below international recommendations, it is comparatively higher within vulnerable populations [18, 19]. To put our findings into context, we compared influenza and pneumococcal vaccination rates with those reported from other countries (Supplementary Table S11). Pneumococcal vaccination rates were relatively consistent across countries, ranging from 30% to 38%, whereas influenza coverage showed greater heterogeneity, ranging from 41% to 87% [16, 20–22]. Although some studies indicate varying rates depending on pathologies [14, 15], disease type did not seem to impact these influenza and pneumococcal vaccination in our study.

Regarding lower pneumococcal vaccination uptake, it is important to emphasize the specificity of the pneumococcal vaccine, which differs from the flu vaccine administered each season. Despite being less often required than the flu, we observe lower reported uptake, which may suggest ignorance or misinformation on this subject and could explain some of these results. Moreover, patients were more confident in answering questions on influenza vaccination (1.5% unknown responses) than when it came to pneumococcus (37% unknown responses) or checking their vaccination status (26% unknown responses). In Switzerland, pneumococcal vaccination consists of a single administration of the conjugated vaccine. As this vaccination is not given annually, recall bias cannot be excluded, particularly for vaccinations received several years earlier. These figures highlight a significant gap in patient knowledge and awareness about pneumococcal vaccination and the importance of maintaining updated vaccination status. Analysis of associated factors indicated that beliefs about the importance of vaccination and discussions with a rheumatologist about vaccination were significantly associated with vaccination uptake. However, positive opinion of the importance of vaccination and discussion about vaccination with their rheumatologist was significantly associated with vaccination uptake.

In our study, patients exhibited strong beliefs in the importance of vaccination, which was significantly associated with verified checking vaccination status within the past 24 months and influenza coverage. A large majority of participants reported that vaccination is important, yet vaccination uptake was relatively low. They, however, show more concerns about vaccine safety, with just over half finding vaccination safe, which may suggest a lack of knowledge. This is in line with the study from Seo *et al.*, which demonstrated that, despite recognizing the importance of vaccination and a sense of security among professionals and patients, only 37% of practitioners surveyed had vaccinated >60% of their patients [12]. This international study identified possible reasons for this, including a lack of time and vaccination-related experience and a lack of knowledge about vaccination guidelines [12].

Interestingly, discussing with rheumatologists regarding vaccination was positively associated with vaccination uptake. However, in our study, only 31% of rheumatologists reported checking their patients’ vaccination status, which is low considering the impact this discussion can have on vaccination intention. These results emphasize the importance of the clinical encounter between patients and their rheumatologist in vaccination decision-making, in line with previous studies [13, 14, 23, 24].

For patients receiving b/tsDMARDs, we observed slightly higher vaccination uptake than those receiving conventional treatment or no treatment, which is inconsistent with other studies indicating that biologic treatments may negatively influence vaccination coverage [4, 17, 25]. We also found a positive association between influenza and pneumococcal coverage and bDMARD treatment, which is consistent with Swiss recommendations that encourage annual influenza vaccination for immunocompromised patients [26].

The main limitation of our study is selection bias. Only individuals who answered the questionnaire were included, which could influence the representativeness of our sample. In addition, we used self-administered questionnaires to collect information. This method could have led to measurement and memory errors and affected the reliability of our data. Moreover, the topic of vaccination itself is sometimes difficult to address and may have led to a social desirability bias and an overestimation of vaccination coverage in our sample. Nevertheless, our results are in line with previous studies showing a relatively low vaccine uptake. Finally, we were not able to adjust for comorbidities, which may have influenced vaccination rates.

Our study has several strengths that reinforce the validity and relevance of our results. First, the size of our sample was important, allowing us to obtain robust and representative data on vaccination behaviours among patients with rheumatic diseases in all of Switzerland. Second, our approach included patients with different rheumatic diseases, providing a more comprehensive and nuanced understanding of factors influencing vaccination decisions in this population. Third, the diversity of medical centres included in our sample enhances the generalizability of our results to other clinical contexts. Last, the sensitivity analysis enhances the reliability and credibility of our results.

Our results emphasize the importance of raising awareness among patients and healthcare professionals about the importance of vaccination in people with rheumatic diseases. Awareness and education campaigns should highlight the benefits of vaccination for the prevention of infectious diseases, as well as the safety of vaccines in this population. Clinicians should systematically incorporate conversations about the importance and safety of vaccination during consultations, particularly when initiating treatment, changing treatment or preparing for travel. Indeed, checking vaccination before initiating or changing treatment could maximize their immunogenicity. Despite some studies demonstrating the safety and effectiveness of vaccines in this population [27–29], the fear of adverse effects still remains an obstacle, particularly among patients who may worry that vaccines will trigger disease flares or interact with their immunosuppressive medications [5, 17, 24, 30]. Directly addressing patients’ concerns about vaccine effectiveness or safety is essential to dispel fears and correct misconceptions. Concerns about vaccine safety and perceived importance can be a major barrier to vaccination and should be the focus of targeted interventions. Health organizations could implement awareness campaigns to inform about the benefits of vaccination for preventing infectious diseases and provide clear guidelines, especially for this specific at-risk population. Regular training for healthcare professionals and improving access to vaccination, notably with reminder programs, can also help increase vaccination uptake in this vulnerable population [31].

In conclusion, vaccination uptake is still too low among patients with AIIRD. Discussions with healthcare professionals and rheumatologists significantly promote vaccination uptake among patients with AIIRD. It is crucial to recognize patient hesitancy towards vaccination and to consider their concerns and beliefs during these discussions to promote informed and shared decision-making and improve overall patient care.

## Supplementary Material

keag123_Supplementary_Data

## Data Availability

This study is based on data collected by the SCQM Foundation (Swiss Clinical Quality Management in Rheumatic Diseases), which operates a national register for inflammatory rheumatic diseases. Access to the data is subject to restrictions and requires approval from the SCQM Foundation in accordance with the SCQM Rules of Research and Collaboration (https://www.scqm.ch/en/research/research-with-scqm-data/). Interested parties may contact the SCQM Foundation (research@scqm.ch) to request access to the data for research purposes.
