# Supplementary material for: Vaccination rates in rheumatic diseases: a cross-sectional register study on the role of patient beliefs and physician engagement
Source: Rheumatology (Oxford). 2026 Mar 15;65(4):keag123. doi: 10.1093/rheumatology/keag123 (PMC13070681; doi:10.1093/rheumatology/keag123)
Supplement: keag123_Supplementary_Data [file keag123_supplementary_data.docx]

***Supplementary Table S1 : Questionnaire***

| Questions | Responses |
| --- | --- |
| Do you think it is important, that everyone get the recommended vaccines for themselves and their children? | - Completely agree - Agree - Neither disagree nor agree - Disagree - Completely disagree |
| Do you think the vaccines are safe for you? | - Extremely safe - Very safe - Fairly safe - Not very safe - Not at all safe |
| Have it been verified that your vaccinations were up to date in the last 24 months? | - Yes - No - I don’t know |
| Did you discuss vaccination with your rheumatologist? | - Yes - No - I don’t know |
| Was it checked that your vaccinations were up to date before the introduction of your current rheumatological treatment? | - Yes - No - I don’t know |
| Have you discussed with a doctor the vaccinations needed for future travel before starting your current rheumatologic treatment? | - Yes - No - I don’t know |
| Since the COVID-19 pandemic, has your desire for vaccination changed? | - More - Equal - Less |
| Have you been vaccinated for seasonal flu this winter (2020-2021)? | - Yes - No - I don’t know |
| Are you vaccinated for : | |
| - Pneumococcal pneumonia - Hepatitis A - Hepatitis B - Measles - Herpes zoster - Yellow fever - Tick encephalitis | - Yes - No - I don’t know |

***Supplementary Table S2: Cross-tabulation of vaccination importance and vaccine safety perception and correlation analysis***

| Variables (n (%)) | Vaccination Safety | | | | | | | Row total | Pearson’s Chi-squared test |
| --- | --- | --- | --- | --- | --- | --- | --- | --- | --- |
| Vaccination Importance |  | | *Not safe* | | *Fairly safe* | | *Safe* |  | *p <0.001* |
|  | *Disagrees* | | 58(2.4) | | 13(0.5) | | 1(0.0) | 72(2.9) |  |
|  | *No opinion* | | 128(5.2) | | 295(12.1) | | 55(2.2) | 478(19.5) |  |
|  | *Agrees* | | 37(1.5) | | 581(23.8) | | 1278(52.2) | 1896(77.5) |  |
| Column total |  | | *223(9.1)* | | 889(36.3) | | 1334(54.5) | 2446 (100) |  |
|  |  |  | |  | |  | |  |  |

***Supplementary Table S3: Population characteristics according to their disease (N (%))***

| Variables | level | Axial spondyloarthritis | | Psoriatic arthritis | | Rheumatoid arthritis |
| --- | --- | --- | --- | --- | --- | --- |
| N = 2446 |  | | N = 942 | N = 604 | | N = 900 |
| Age | ≤65 | | 844 (90) | 491 (81) | | 607 (67) |
|  | >65 | | 98 (10) | 113 (19) | | 293 (33) |
| Gender | Female | | 480 (51) | 312 (52) | | 663 (74) |
|  | Male | | 462 (49) | 292 (48) | | 237 (26) |
| Body Mass Index | Normal weight | | 406 (47) | 193 (35) | | 406 (49) |
|  | Pre-obesity | | 304 (35) | 201 (36) | | 268 (33) |
|  | Obesity | | 147 (17) | 162 (29) | | 150 (18) |
|  | NA^1^ | | 85 | 48 | | 76 |
| Disease duration  (median [Q1,Q3]) |  | | 11 [5,17] | | 10 [5,16] | 11 [6,18] |
| Treatments | Biological DMARD | | 679 (72) | 453 (75) | | 626 (70) |
|  | Conventional DMARD | | 16 (1.7) | 61 (10) | | 183 (20) |
|  | No treatment | | 247 (26) | 90 (15) | | 91 (10) |
| Glucocorticoids | No | | 930 (99) | 579 (96) | | 786 (87) |
|  | Yes | | 12 (1.3) | 25 (4.1) | | 114 (13) |
| ^1^ Not available |  | |  |  | |  |

***Supplementary Table S4: Multivariate analysis of factors associated with checking the vaccination status in the last 24 months***

| Variables | Levels | OR^1^ | 95% CI^2^ | P-value |
| --- | --- | --- | --- | --- |
| Vaccination importance | Disagrees | Ref |  |  |
|  | No opinion | 1.23 | 0.61, 2.49 | 0.6 |
|  | Agrees | 2.07 | 1.03, 4.16 | 0.042 |
| Age | ≤65 | Ref |  |  |
|  | >65 | 1.56 | 1.21, 2.03 | <0.001 |
| Gender | Female | Ref |  |  |
|  | Male | 0.86 | 0.70, 1.06 | 0.2 |
| Body Mass Index | Normal weight | Ref |  |  |
|  | Pre-obesity | 1.13 | 0.89, 1.43 | 0.3 |
|  | Obesity | 1.36 | 1.02, 1.81 | 0.037 |
| Disease | Rheumatoid arthritis | Ref |  |  |
|  | Axial spondyloarthrits | 0.93 | 0.72, 1.19 | 0.6 |
|  | Psoriatic arthritis | 0.76 | 0.58, 1.00 | 0.048 |
| Disease duration |  | 0.98 | 0.97, 0.99 | 0.002 |
| Treatments | No treatment | Ref |  |  |
|  | Conventional DMARD | 0.82 | 0.55, 1.24 | 0.3 |
|  | Biological DMARD | 0.79 | 0.59, 1.05 | 0.10 |
| Glucocorticoids | No | Ref |  |  |
|  | Yes | 0.86 | 0.57, 1.31 | 0.5 |
| Vaccination discussed with rheumatologist | No | Ref |  |  |
|  | Yes | 1.35 | 1.01, 1.79 | 0.041 |
| Vaccination status verified before treatment | No | Ref |  |  |
|  | Yes | 10.8 | 8.61, 13.5 | <0.001 |
| Vaccination discussed travel requirements | No | Ref |  |  |
|  | Yes | 2.64 | 2.09, 3.33 | <0.001 |
| Vaccination desire since COVID-19 | Less | Ref |  |  |
|  | Equal | 1.29 | 0.95, 1.74 | 0.10 |
|  | More | 1.40 | 0.97, 2.03 | 0.072 |
| ^1^ Odds Ratio, ^2^ Confidence Interval | | | | |

***Supplementary Table S5: Multivariate analysis of factors associated with influenza vaccination coverage***

| Variables | Levels | OR^1^ | 95% CI^2^ | P-value |
| --- | --- | --- | --- | --- |
| Vaccination importance | Disagrees | Ref |  |  |
|  | No opinion | 1.45 | 0.72, 2.91 | 0.3 |
|  | Agrees | 4.01 | 2.01, 7.98 | <0.001 |
| Age | ≤65  >65 | Ref  1.97 | 1.56,2.48 | <0.001 |
| Gender | Female | Ref |  |  |
|  | Male | 0.71 | 0.59, 0.85 | <0.001 |
| Body Mass Index | Normal weight | Ref |  |  |
|  | Pre-obesity | 1.10 | 0.90, 1.36 | 0.3 |
|  | Obesity | 1.20 | 0.94, 1.54 | 0.14 |
| Disease | Rheumatoid arthritis | Ref |  |  |
|  | Axial spondyloarthritis | 1.00 | 0.80, 1.25 | >0.9 |
|  | Psoriatic arthritis | 1.01 | 0.80, 1.28 | >0.9 |
| Disease duration |  | 1.01 | 1.01, 1.02 | 0.002 |
| Treatments | No treatment | Ref |  |  |
|  | Conventional DMARD | 1.06 | 1.27 | 0.89, 1.81 |
|  | Biological DMARD | 2.02 | 1.58, 2.59 | <0.001 |
| Glucocorticoids | No | Ref |  |  |
|  | Yes | 1.36 | 0.94, 1.99 | 0.10 |
| Vaccination discussed with rheumatologist | No | Ref |  |  |
|  | Yes | 1.27 | 1.00, 1.61 | 0.051 |
| Vaccination status verified before treatment | No | Ref |  |  |
|  | Yes | 1.37 | 1.13, 1.66 | 0.001 |
| Vaccination discussed travel requirements | No | Ref |  |  |
|  | Yes | 1.03 | 0.83, 1.26 | 0.8 |
| Vaccination desire since COVID-19 | Less | Ref |  |  |
|  | Equal | 2.13 | 1.63, 2.78 | <0.001 |
|  | More | 2.49 | 1.80, 3.45 | <0.001 |
| ^1^ Odds Ratio, ^2^ Confidence Interval | | | | |

***Supplementary Table S6: Multivariate analysis of factors associated with pneumococcus vaccination uptake***

| Variables | Levels | OR^1^ | 95% CI^2^ | P-value |
| --- | --- | --- | --- | --- |
| Vaccination importance | Disagrees | Ref |  |  |
|  | No opinion | 1.13 | 0.57, 2.25 | 0.7 |
|  | Agrees | 1.79 | 0.91, 3.53 | 0.094 |
| Age | ≤65  >65 | Ref  0.83 | 0.66, 1.05 | 0.15 |
| Gender | Female | Ref |  |  |
|  | Male | 0.49 | 0.40, 0.59 | <0.001 |
|  |  |  |  |  |
| Body Mass Index | Normal weight | Ref |  |  |
|  | Pre-obesity | 1.02 | 0.82, 1.26 | 0.9 |
|  | Obesity | 1.07 | 0.83, 1.38 | 0.6 |
| Disease | Rheumatoid arthritis | Ref |  |  |
|  | Axial spondyloarthritis | 1.20 | 0.96, 1.51 | 0.11 |
|  | Psoriatic arthritis | 1.07 | 0.84, 1.36 | 0.6 |
| Disease duration |  | 0.99 | 0.98, 1.00 | 0.006 |
| Treatments | No treatment | Ref |  |  |
|  | Conventional DMARD | 1.09 | 0.74, 1.59 | 0.7 |
|  | Biological DMARD | 1.39 | 1.07, 1.81 | 0.014 |
| Glucocorticoids | No | Ref |  |  |
|  | Yes | 1.08 | 0.75, 1.56 | 0.7 |
| Vaccination discussed with rheumatologist | No | Ref |  |  |
|  | Yes | 1.33 | 1.02, 1.74 | 0.035 |
| Vaccination status verified before treatment | No | Ref |  |  |
|  | Yes | 2.19 | 1.78, 2.69 | <0.001 |
| Vaccination discussed travel requirements | No | Ref |  |  |
|  | Yes | 1.47 | 1.20, 1.80 | <0.001 |
| Vaccination desire since COVID-19 | Less | Ref |  |  |
|  | Equal | 1.41 | 1.06, 1.87 | 0.018 |
|  | More | 1.27 | 0.91, 1.79 | 0.2 |
| ^1^ Odds Ratio, ^2^ Confidence Interval | | | | |

**Sensitivity analysis**

***Supplementary Table S7: Multivariate analysis of factors associated with update of the vaccination status in the last 24 months with uncertain responses coded as “Missing data”***

| Variables | Levels | OR^1^ | 95% CI^2^ | P-value |
| --- | --- | --- | --- | --- |
| Vaccination importance | Disagrees | Ref |  |  |
|  | No opinion | 1.31 | 0.51, 3.38 | 0.6 |
|  | Agrees | 2.11 | 0.80, 5.57 | 0.13 |
| Age | ≤65 | Ref |  |  |
|  | >65 | 1.53 | 1.07, 2.19 | 0.021 |
| Gender | Female | Ref |  |  |
|  | Male | 0.95 | 0.73, 1.23 | 0.7 |
| Body Mass Index | Normal weight | Ref |  |  |
|  | Pre-obesity | 1.14 | 0.86, 1.51 | 0.4 |
|  | Obesity | 1.27 | 0.92, 1.77 | 0.15 |
| Disease | Rheumatoid arthritis | Ref |  |  |
|  | Axial spondyloarthritis | 0.86 | 0.63, 1.18 | 0.4 |
|  | Psoriatic arthritis | 0.68 | 0.48, 0.95 | 0.023 |
| Disease duration |  | 0.98 | 0.97, 1.00 | 0.015 |
| Treatments | No treatment | Ref |  |  |
|  | Conventional DMARD | 1.09 | 0.65, 1.83 | 0.7 |
|  | Biological DMARD | 0.77 | 0.53, 1.10 | 0.15 |
| Glucocorticoids | No | Ref |  |  |
|  | Yes | 1.07 | 0.63, 1.81 | 0.8 |
| Vaccination discussed with rheumatologist | No | Ref |  |  |
|  | Yes | 1.00 | 0.65, 1.54 | >0.9 |
| Vaccination status verified before treatment | No | Ref |  |  |
|  | Yes | 17.7 | 12.0, 26.3 | <0.001 |
| Vaccination discussed travel requirements | No | Ref |  |  |
|  | Yes | 3.46 | 2.52, 4.74 | <0.001 |
| Impact of COVID | Less | Ref |  |  |
|  | Equal | 1.17 | 0.80, 1.72 | 0.4 |
|  | More | 1.22 | 0.78, 1.91 | 0.4 |
| ^1^ Odds Ratio, ^2^ Confidence Interval |  |  |  |  |

***Supplementary Table S8: Multivariate analysis of factors associated with influenza vaccination coverage with uncertain responses coded as “Missing data”***

| Variables | Levels | OR^1^ | 95% CI^2^ | P-value |
| --- | --- | --- | --- | --- |
| Vaccination importance | Disagrees | Ref |  |  |
|  | No opinion | 1.35 | 0.66, 2.78 | 0.4 |
|  | Agrees | 3.71 | 1.84, 7.50 | <0.001 |
| Age | ≤65 | Ref |  |  |
|  | >65 | 1.97 | 1.56, 2.49 | <0.001 |
| Gender | Female | Ref |  |  |
|  | Male | 0.71 | 0.59, 0.86 | <0.001 |
| Body Mass Index | Normal weight | Ref |  |  |
|  | Pre-obesity | 1.13 | 0.91, 1.39 | 0.3 |
|  | Obesity | 1.25 | 0.98, 1.60 | 0.070 |
| Disease | Rheumatoid arthritis | Ref |  |  |
|  | Axial spondyloarthritis | 0.98 | 0.79, 1.23 | 0.9 |
|  | Psoriatic arthritis | 0.99 | 0.78, 1.26 | >0.9 |
| Disease duration |  | 1.01 | 1.00, 1.02 | 0.004 |
| Treatments | No treatment | Ref |  |  |
|  | Conventional DMARD | 1.24 | 0.87, 1.77 | 0.2 |
|  | Biological DMARD | 2.00 | 1.56, 2.57 | <0.001 |
| Glucocorticoids | No | Ref |  |  |
|  | Yes | 1.44 | 0.97, 2.11 | 0.068 |
| Vaccination discussed with rheumatologist | No | Ref |  |  |
|  | Yes | 1.16 | 0.88, 1.55 | 0.3 |
| Vaccination status verified before treatment | No | Ref |  |  |
|  | Yes | 1.48 | 1.13, 1.94 | 0.004 |
| Vaccination discussed travel requirements | No | Ref |  |  |
|  | Yes | 1.13 | 0.91, 1.41 | 0.3 |
| Impact of COVID | Less | Ref |  |  |
|  | Equal | 2.18 | 1.66, 2.85 | <0.001 |
|  | More | 2.51 | 1.81, 3.48 | <0.001 |
| ^1^ Odds Ratio, ^2^ Confidence Interval |  |  |  |  |

***Supplementary Table S9: Multivariate analysis of factors associated with pneumococcal vaccination coverage with uncertain responses coded as “Missing data”***

| Variables | Levels | | | OR^1^ | 95% CI^2^ | | P-value |  |
| --- | --- | --- | --- | --- | --- | --- | --- | --- |
| Vaccination importance | Disagrees | | | Ref |  | |  |  |
|  | No opinion | | | 1.10 | 0.43, 2.80 | | 0.8 |  |
|  | Agrees | | | 1.86 | 0.77, 4.53 | | 0.2 |  |
| Age | ≤65 | | | Ref |  | |  |  |
|  | >65 | | | 0.75 | 0.58, 0.99 | | 0.039 |  |
| Gender | Female | | | Ref |  | |  |  |
|  | Male | | | 0.58 | 0.47, 0.72 | | <0.001 |  |
| Body Mass Index | Normal weight | | | Ref |  | |  |  |
|  | Pre-obesity | | | 0.99 | 0.78, 1.25 | | >0.9 |  |
|  | Obesity | | | 1.00 | 0.73, 1.37 | | >0.9 |  |
| Disease | Rheumatoid arthritis | | | Ref |  | |  |  |
|  | Axial spondyloarthritis | | | 1.11 | 0.83, 1.47 | | 0.5 |  |
|  | Psoriatic arthritis | | | 1.05 | 0.79, 1.39 | | 0.7 |  |
| Disease duration |  | | | 0.98 | 0.97, 1.00 | | 0.009 |  |
| Treatments | No treatment | | | Ref |  | |  |  |
|  | Conventional DMARD | | | 0.79 | 0.49, 1.26 | | 0.3 |  |
|  | Biological DMARD | | | 1.17 | 0.80, 1.70 | | 0.4 |  |
| Glucocorticoids | No | | | Ref |  | |  |  |
|  | Yes | | | 0.79 | 0.79 | | 0.79 |  |
| Vaccination discussed with rheumatologist | No | | | Ref |  | |  |  |
|  | Yes | | | 1.16 | 0.74, 1.82 | | 0.5 |  |
| Vaccination status verified before treatment | No | | | Ref |  | |  |  |
|  | Yes | | | 2.70 | 1.89, 3.86 | | <0.001 |  |
| Vaccination discussed travel requirements | No | | | Ref |  | |  |  |
|  | Yes | | | 1.63 | 1.27, 2.10 | | <0.001 |  |
| Impact of COVID | Less | | | Ref |  | |  |  |
|  | Equal | | | 1.44 | 1.06, 1.96 | | 0.021 |  |
|  | More | | | 1.17 | 0.81, 1.68 | | 0.4 |  |
| ^1^ Odds Ratio, ^2^ Confidence Interval | |  |  | | |  | | |

***Supplementary Table S10 : Percentage of influenza vaccination coverage in the present study and in a Swiss study in the general population between 2007-2017***

|  | ***Present study*** | ***Swiss study 2007-2017*** |
| --- | --- | --- |
| ***Overall population*** | *51%* | *16%* |
| ***>65 years old*** | *67%* | *36-48%* |

***Supplementary Table S11: Comparison of vaccination uptake rates***

| *Country* | *Population (Disease)* | *N* | *Influenza coverage (%)* | *Pneumococcal coverage (%)* | *Reference* |
| --- | --- | --- | --- | --- | --- |
| *Switzerland* | ***AIIRD^1^*** | ***2446*** | ***51*** | ***33*** | ***Present study*** |
| *United Kingdom* | ***AIIRD^1^*** | ***906*** | ***63*** | ***35*** | ***[20]*** |
| *Germany* | ***AIIRD^1^*** | ***222*** | ***69*** | ***35*** | ***[17]*** |
| *France* | ***AIIRD^1^*** | ***406 156*** | ***41*** | ***38*** | ***[19]*** |
| *Japan* | ***AIIRD^1^*** | ***1637*** | ***87*** | ***30*** | ***[21]*** |
| *^1^* *^autoimmune inflammatory rheumatic disease ;^* | | |  |  |  |
